# *ABCB1* polymorphism is associated with atorvastatin-induced liver injury in Japanese population

**DOI:** 10.1186/s12863-016-0390-5

**Published:** 2016-06-13

**Authors:** Koya Fukunaga, Hiroshi Nakagawa, Toshihisa Ishikawa, Michiaki Kubo, Taisei Mushiroda

**Affiliations:** Laboratory for Pharmacogenomics, RIKEN Center for Integrative Medical Sciences, Yokohama, Japan; Department of Biological Chemistry, College of Bioscience and Biotechnology, Chubu University, Aichi, Japan; NPO Personalized Medicine & Healthcare, Yokohama, Japan; Laboratory for Genotyping Development, RIKEN Center for Integrative Medical Sciences, Yokohama, Japan

**Keywords:** Atorvastatin-induced adverse reaction, Genetic association, Hepatotoxicity, MDR1 Ala893Ser/Thr/

## Abstract

**Background:**

To investigate the associations between atorvastatin-induced liver injury (AILI) and polymorphisms in eight genes possibly involved in the hepatic metabolism (*CYP2C9*, *CYP2C19*, *CYP3A4*, *CYP3A5* and *UGT1A1*) and membrane transport (*ABCB1*, *ABCG2* and *SLCO1B1*) of atorvastatin, we genotyped 30 AILI and 414 non-AILI patients recruited at BioBank Japan for 15 single nucleotide polymorphisms (SNPs).

**Results:**

An SNP in *ABCB1* (rs2032582: 2677G > T/A) was significantly associated with AILI (*P* = 0.00068, odds ratio (OR) = 2.59 with 95 % confidence interval (CI) of 1.49-4.50, G allele versus T and A alleles), indicating that the G allele might be a risk factor for AILI. The cytotoxicity test demonstrated that IC_50_ value of atorvastatin to inhibit the growth and/or viability of Flp-In-293/ABCB1 (2677G) cells was 5.44 ± 0.10 mM, which was significantly lower than those in Flp-In-293/ABCB1 (2677 T) (6.02 ± 0.07 mM) and Flp-In-293/ABCB1 (2677A) cells (5.95 ± 0.08 mM).

**Conclusions:**

These results indicate that *ABCB1* rs2032582 may predict the risk of AILI in Japanese population.

**Electronic supplementary material:**

The online version of this article (doi:10.1186/s12863-016-0390-5) contains supplementary material, which is available to authorized users.

## Background

Atorvastatin (atorvastatin calcium; Lipitor®) is widely used in the treatment of dyslipidemia of low- and high-density lipoproteins in patients with or without heart disease [1]. However, atorvastatin-induced liver injury (AILI) can be caused after atorvastatin treatment [1, 2]. In Japanese post-marketing surveillance of atorvastatin, 1.42 % of patients who received atorvastatin treatment suffered from liver injury. In general, drug-induced liver injury (DILI) can be divided into 3 types (hepatocellular injury, cholestatic liver injury and mixed liver injury) based on potential liver toxicity symptoms (e.g., anorexia, nausea, vomiting or jaundice), the presence or absence of risk factors (e.g., viral infection and alcohol consumption) and serum levels of alanine aminotransferase (ALT) and alkaline phosphatase (ALP) as well as the ALT/ALP ratio [3]. AILI falls within the hepatocellular injury category because ALT level of two patients treated with atorvastatin reportedly raised three-fold higher than that of the upper limit of normal but ALP and bilirubin levels did not change [4].

Atorvastatin is orally administered in the active acid form and undergoes marked first-pass metabolism by uptake into hepatocytes via passive diffusion and *SLCO1B1* (encoding OATP1B1 [5–7]. Atorvastatin is metabolized mainly by *CYP3A4*, with minor contributions from *CYP2C9*, *CYP2C19*, *CYP3A5*, and *UGT1A1* [8–12]. Subsequently, atorvastatin and the metabolites are predominantly eliminated by *ABCB1* (encoding P-glycoprotein or MDR1)- and *ABCG2* (encoding BCRP)-mediated transport from liver into bile [7, 13–15]. Single nucleotide polymorphisms (SNPs) identified in *ABCB1* rs1128503 (1236C > T), rs2032582 (2677G > T/A), and rs1045642 (3435C > T) markedly affected area under the plasma concentration versus time curve (AUC) of atorvastatin and the lipid-lowering effects of atorvastatin therapy [16–18]. Therefore, we hypothesized that the genetic variability of eight candidate genes associated with the hepatic metabolism and membrane transport of atorvastatin may affect the risk of AILI because higher concentrations of atorvastatin can cause hepatocellular injury, even at appropriate atorvastatin dosages. However, to our knowledge, there are no reports on an association of the functional SNPs of the candidate genes with AILI.

In this study, we investigated whether 15 functional SNPs in eight candidate genes that are possibly involved in the pharmacokinetics of atorvastatin were associated with AILI in Japanese population. We found that *ABCB1* rs2032582 was significantly associated with AILI. In addition, the cytotoxicity of atorvastatin was investigated using the Flp-In-293 cells stably expressing ABCB1 proteins encoded by *ABCB1* rs2032582 [19]. We clarified that the *ABCB1* rs2032582 G allele was a significant AILI risk factor i*n vivo* and *in vitro.*

## Methods

### Subjects

The BioBank Japan project (https://biobankjp.org/) started in 2003 for the collection of genomic DNA, serum and clinical information from about 300,000 Japanese patients diagnosed with either of 47 diseases by a collaborative network of 66 hospitals in Japan. We diagnosed AILI based on symptoms, such as nausea, vomiting, loss of appetite, and jaundice, and results of a physical examination and blood tests after atorvastatin administration. From the registered samples in the BioBank Japan, we selected individuals that were clinically diagnosed as having AILI (AILI group, *N* = 30) and individuals that showed no liver injury during atorvastatin therapy (non-AILI group, *N* = 414).

### Selection of SNPs and genotyping

A total of 15 functional SNPs in eight candidate genes (*ABCB1*, *ABCG2*, *CYP2C9*, *CYP2C19*, *CYP3A4*, *CYP3A5*, *SLCO1B1* and *UGT1A1*) reportedly-altering pharmacokinetics of atorvastatin were genotyped by a multiplex polymerase chain reaction (PCR)–based invader assay as described previously [20] and direct sequencing using ABI 3730xl DNA analyzer (Applied Biosystems, Foster City, CA) for rs8175347 and rs2032582, according to the manufacturer’s protocol of the Big Dye Terminator v3.1 cycle sequencing kit (Applied Biosystems). HLA-A, -B and -C genotyping was carried out using a WAKFlow HLA Typing kit (Wakunaga, Osaka, Japan), which is based on PCR-sequence-specific oligonucleotide probes coupled with multiple analyte profiling (xMAP) technology (Luminex System; Luminex Corporation, Austin, TX). The data analysis was performed using the WAKFlow Typing software (Wakunaga).

### Cell culture

HepaRG cells (KAC, Kyoto, Japan) were maintained in HepaRG Thawing and Seeding Medium 670 (KAC) and HepaRG Maintenance and Metabolism medium 620 (KAC) at 37 °C under 5 % CO_2_ and 95 % air according to the manufacturer’s instructions. Flp-In-293 cells (Life Technologies, Foster City, CA) were maintained in Dulbecco’s modified Eagle’s medium (Life Technologies) supplemented with 10 % heat-inactivated fetal bovine serum (Life Technologies) and Antibiotic-Antimycotic (100×) liquid (Life Technologies) at 37 °C under 5 % CO_2_ and 95 % air, where 100 μg/ml Zeocin (Life Technologies) and 100 μg/ml hygromycin B (Life Technologies) were also supplemented for the maintenance for parental and ABCB1-expressed Flp-In-293 cells, respectively.

### Generation of *ABCB1* 2677G/T/A variant forms

The pcDNA5/FRT/ABCB1 (2677G) vector was generated by inserting *ABCB1* (2677G) in pFastBac1/ABCB1 (2677G) into the pcDNA5/FRT vector (Life Technologies) between the restriction enzyme sites of *BamH I* and *Xho I. ABCB1* 2677 T/A variant forms were generated by using the QuikChange Site-directed Mutagenesis Kit (Agilent Technologies, Santa Clara, CA) according to the manufacturer’s protocol, where pcDNA5/FRT/ABCB1 (2677G) vector was used as the template [21]. The PCR reaction consisted of 94 °C for 2 min and then followed by 12 cycles of reactions at 94 °C for 30 sec, 55 °C for 30 sec and at 68 °C for 18 min, where Pfu Turbo DNA polymerase (Agilent Technologies) and the following PCR primers were used: 5’-GAAAGAACTAGAAGGTTCTGGGAAGATCGCTAC-3’ and 5’-GTAGCGATCTTCCCAGAACCTTCTAGTTCTTTC-3’ for *ABCB1* 2677 T, and 5’-GAAAGAACTAGAAGGTACTGGGAAGATCGCTAC-3’ and 5’-GTAGCGATCTTCCCAGTACCTTCTAGTTCTTTC-3’ for *ABCB1* 2677A. After the PCR, the reaction mixture was incubated with *Dpn*I endonuclease at 37 °C for 1 h to digest the original template pcDNA5/FRT/ABCB1 (2677G) vector. The resulting sequence was examined to confirm the generation of the pcDNA5/FRT/ABCB1 (2677G/T/A) vectors.

### Establishment of *ABCB1* 2677G/T/A variant forms-expressing cells

Flp-In-293 cells having the Flp Recombination Target (FRT) site at the telomeric region of only one of the pair of chromosomes 12 were transfected with the pcDNA5/FRT/ABCB1 (2677G/T/A) and the Flp recombinase expression plasmid pOG44 vectors as previously reported [19]. Single colonies resistant to hygromycin B (Life Technologies) were picked and sub-cultured as Flp-In-293/ABCB1 (2677G), Flp-In-293/ABCB1 (2677 T) and Flp-In-293/ABCB1 (2677A) cells. Protein expression levels of ABCB1 in these cells (2 × 10^7^ cells) were determined using Membrane Protein Extraction Kit (BioVision, Milpitas, CA) and Human permeability glycoprotein (P-gp/ABCB1) ELISA kit (Cusabio Biotech, Wuhan, China) by a microplate reader (ARVOmx, PerkinElmer, Waltham, MA).

### Cytotoxicity studies

In atorvastatin (Sigma-Aldrich, St Louis, Mo) cytotoxicity experiment, HepaRG (5 × 10^5^ cells/well), Flp-In-293/ABCB1 (2677G), Flp-In-293/ABCB1 (2677 T) and Flp-In-293/ABCB1 (2677A) cells were cultured in monolayers at 37 °C for 24 h in 24-well collagen type I-coated plates (Iwaki Glass, Chiba, Japan). After the preculture, cells were cultured in the presence of different concentrations of atorvastatin 0, 0.3, 1, 3, 10, 30, 100 and 300 μM for HepaRG cells, 0, 0.6, 1, 2, 3, 4, 5, 6, 7, 8, 9 and 10 mM for Flp-In-293/ABCB1 (2677G/T/A) cells for 24 h. After the culture, 50 μl of WST-8 working solution (Cell Counting Kit-8, Dojindo Laboratories, Kumamoto, Japan) was added to each well and the plates were incubated at 37 °C for 1 h under 5 % CO_2_ and 95 % air. Optical density at 450 nm was measured by a microplate reader (ARVOmx, PerkinElmer, Waltham, MA). Lactate dehydrogenase (LDH), aspartate aminotransferase (AST) and ALT releases from HepaRG cells into the medium were determined according to the manufacturers’ protocol of LDH, AST and ALT activity assay kits (Biovision, Milpitas, CA).

### Statistical analysis

Association studies were conducted by using Fisher’s exact test under an allelic model. P values were corrected according to Bonferroni correction. A significance level was set at 0.0029 (0.05/17) in Table 1. In case of *ABCB1* rs2032582, the patients were divided into two groups (T/A versus G, G/A versus T or G/T versus A) to evaluate the association of the three alleles by using the Fisher’s exact test (Table 1). The haplotype analysis was performed using SNPAlyze software (version. 8.0.1, Dynacom, Chiba, Japan). Statistical analysis of cytotoxicity test of HepaRG and ABCB1 protein expression levels in Flp-In-293 cells was performed by using one-way analysis of variance with Dunnett’s and Tukey’s post-hoc test using GraphPad Prism software (version 6, San Diego, CA). Cell viability was analyzed based on four independent experiments performed in duplicate to accurately estimate IC_50_ and statistical analysis of IC_50_ among three groups (2677G wild-type, 2677 T and 2677A alleles) was performed by using one-way analysis of variance with Dunnett’s post-hoc test using GraphPad Prism software.

**Table 1:** Association of 15 functional SNPs in eight candidate genes with atorvastatin-induced liver injury

Gene	SNP	Allele (1/2)	Amino acid change	Other name	AILI^a^			Non-AILI^a^			RAF		P value^b^			HWE	
					11	12	22	11	12	22	AILI	Non-AILI	ALLELIC	DOM	REC	AILI	Non-AILI
*ABCB1*	rs1045642	C/T	I1145I	C3435T	12	15	3	151	196	67	0.65	0.60	0.50	0.70	0.60	0.59	0.80
	rs2032582	T, A/G	S,T893A	G2677 T/A	2	16	12	138	191	85	0.67	0.44	**0.00068**	0.0017	0.020	0.27	0.21
		G, A/T	A,T893S		15	14	1	158	193	63	0.73	0.61	0.073	0.24	0.10	0.29	0.75
		G, T/A	A,S893T		26	4	0	283	114	17	0.93	0.82	0.031	0.039	0.62	0.70	0.21
	rs1128503	T/C	G412G	C1236T	15	12	3	146	197	71	0.70	0.59	0.10	0.12	0.45	0.79	0.74
*ABCG2*	rs2231142	C/A	Q141K	C421A	12	15	3	195	180	39	0.35	0.31	0.57	0.57	0.76	0.59	0.78
*CYP2C9*	rs1799853	C/T	R144C	CYP2C9*2	30	0	0	412	0	0	1.00	1.00	1.00	1.00	1.00	1.00	1.00
	rs1057910	A/C	I359L	CYP2C9*3	28	2	0	393	20	0	0.03	0.02	0.66	0.65	1.00	0.85	0.61
*CYP2C19*	rs4244285	G/A	P227P	CYP2C19*2	16	9	5	197	180	36	0.32	0.31	0.88	0.58	0.18	0.09	0.57
	rs4986893	G/A	W212X	CYP2C19*3	27	3	0	321	86	6	0.95	0.88	0.14	0.16	1.00	0.77	0.93
*CYP3A4*	rs12721627	C/G	T185S	CYP3A4*16	29	1	0	405	8	0	0.02	0.01	0.47	0.47	1.00	0.93	0.84
	rs28371759	T/C	L292P	CYP3A4*18	29	1	0	399	14	0	0.98	0.98	1.00	1.00	1.00	0.93	0.73
*CYP3A5*	rs776746	G/A	–	CYP3A5*3	18	11	1	223	165	26	0.78	0.74	0.54	0.57	1.00	0.66	0.54
*SLCO1B1*	rs2306283	G/A	N130D	SLCO1B1*1B	14	12	4	185	179	50	0.67	0.66	1.00	0.85	0.77	0.58	0.51
	rs4149056	T/C	V174A	SLCO1B1*5	18	11	1	306	95	13	0.22	0.15	0.14	0.13	1.00	0.66	0.10
*UGT1A1*	rs4148323	G/A	G71R	UGT1A1*6	25	5	0	281	118	15	0.92	0.82	0.075	0.10	0.61	0.62	0.55
	rs8175347	(TA)6/(TA)7	–	UGT1A1*28	23	7	0	332	76	6	0.12	0.11	0.83	0.64	1.00	0.47	0.49

Abbreviation: *AILI* atorvastatin-induced liver injury, *RAF* risk allele frequency, *ALLELIC* Allelic model, *Dom* Dominant model, *REC* Recessive model, *HWE* Hardy–Weinberg equilibrium

^a^AILI, *N* = 30; Non-AILI, *N* = 414

^b^The lowest significant P value after Bonferroni correction among three models is shown in bold (*P* < 0.0011)

## Results

No significant association of disease background was observed between AILI and non-AILI patients (Additional file 1: Table S1). The median age values were 61 years (range 27–82) and 66 years (32–89) in AILI and non-AILI groups, respectively. The 60.0 and 53.9 % were male in AILI and non-AILI groups, respectively. All SNPs met quality control criteria (call rate > 95 %, Hardy–Weinberg equilibrium P value > 10^-3^ and minor allele frequency > 1 %). *ABCB1* rs2032582 was found to be associated with an increased risk of AILI (*P* = 0.00068, odds ratio (OR) = 2.59 with 95 % confidence interval (CI) of 1.49–4.50, G allele versus T and A alleles) by genotyping 444 Japanese subjects for 15 functional SNPs in eight candidate genes that reportedly affect the pharmacokinetics of atorvastatin (Table 1). No other polymorphisms showed a significant association with AILI. The frequency for *ABCB1* rs2032582 G allele in AILI patients was significantly higher than that in non-AILI patients whereas the frequencies of *ABCB1* rs2032582 T and A alleles were not significantly different between AILI and non-AILI groups, indicating that the G allele might be a risk factor for AILI (Table 1 and Additional file 1: Table S2). Although we performed haplotype analysis using three SNPs of *ABCB1* (rs1128503, rs2032582 and rs1045642), no haplotype constructed from the SNPs showed an extremely smaller P value than a single marker association of the *ABCB1* rs2032582 (Table 2). No association of HLA-A, -B and -C genotypes with AILI was shown (Additional file 1: Table S3, Additional file 1: Table S4 and Additional file 1: Table S5).

**Table 2:** Association of haplotypes consisting of three SNPs of *ABCB1* with atorvastatin-induced liver injury

rs1128503	rs2032582	rs1045642	Number of carriers		P value^b^
			AILI^a^ (%)	Non-AILI^a^ (%)	
T	T	T	15 (50.0)	237 (57.2)	0.45
C	G	C	11 (36.7)	158 (38.2)	1.00
T	G	C	16 (53.3)	126 (30.4)	0.014
C	A	C	4 (13.3)	128 (30.9)	0.060
T	G	T	3 (10.0)	25 (6.0)	0.45
T	T	C	0 (0.0)	24 (5.8)	0.39
C	G	T	2 (6.7)	11 (2.7)	0.22
T	A	C	0 (0.0)	5 (1.2)	1.00
C	T	T	0 (0.0)	2 (0.5)	1.00
C	T	C	0 (0.0)	2 (0.5)	1.00

Abbreviation: *AILI* atorvastatin-induced liver injury

^a^AILI, *N* = 30; Non-AILI, *N* = 414

^b^The significant P value after Bonferroni correction is less than 0.005

The cytotoxicity study using HepaRG cells demonstrated concentration-dependent effects of atorvastatin on cell viability as well as on LDH, AST and ALT release from the cells (Additional file 1: Figure S1). To estimate the effects of *ABCB1* rs2032582 on cytotoxicity induced by atorvastatin, we conducted cytotoxicity study using Flp-In-293 cells stably expressing ABCB1 proteins encoded by 2677G wild-type [Flp-In-293/ABCB1 (2677G) cells], 2677A [Flp-In-293/ABCB1 (2677A) cells] and 2677 T [Flp-In-293/ABCB1 (2677 T) cells] alleles. No significant differences were observed in ABCB1 protein expression levels in Flp-In-293/ABCB1 (2677G/T/A) cells (Additional file 1: Figure S2). The IC_50_ value in Flp-In-293/Mock cells was about two-fold lower than those in Flp-In-293/ABCB1 (2677G/T/A) cells, indicating higher accumulation of atorvastatin in the Flp-In-293/Mock cells compared to that in Flp-In-293/ABCB1 (2677G/T/A) cells. The IC_50_ value in Flp-In-293/ABCB1 (2677G) cells was significantly lower than those in Flp-In-293/ABCB1 (2677 T) and Flp-In-293/ABCB1 (2677A) cells (Table 3, Additional file 1: Figure S3).

**Table 3:** Atorvastatin-dependent cytotoxicity in Flp-In-293 cells stably expressing different ABCB1 proteins

Cell name	ABCB1		IC_50_ (mM)	P value
	Allele	Amino acid	Mean ± SE	
Mock	–	–	2.74 ± 0.04	–
2677G wild-type	G	Alanine	5.44 ± 0.10	–
2677 T	T	Serine	6.02 ± 0.07	**0.009**
2677A	A	Threonine	5.95 ± 0.08	**0.026**

Abbreviation: *SE* standard error, *CI* confidence interval

Experiments were performed in duplicate wells and repeated four times. The significant P value is shown in bold (*P* < 0.05, versus 2677G wild-type, one-way analysis of variance with Dunnett’s post-hoc test)

## Discussion

To identify the genetic markers associated with AILI, we genotyped 15 functional SNPs in eight genes that are possibly involved in the hepatic metabolism (*CYP2C9*, *CYP2C19*, *CYP3A4*, *CYP3A5* and *UGT1A1*) and membrane transport (*ABCB1*, *ABCG2* and *SLCO1B1*) of atorvastatin. *ABCB1* rs2032582 was significantly associated with AILI. *ABCB1* rs2032582 changes ABCB1 amino acid 893 from alanine to serine or threonine, respectively. These variants did not appear to affect ABCB1 protein expression levels in Flp-In-293/ABCB1 (2677G/T/A) cells, but gave a lower IC_50_ in Flp-In-293/ABCB1 (2677G) cells than those in Flp-In-293/ABCB1 (2677 T/A) cells. ATP-dependent uptake of [^3^H]-vincristine into membrane vesicles is also reportedly slower in cells expressing the *ABCB1* rs2032582 G allele than those expressing the *ABCB1* rs2032582 T/A alleles [22]. Therefore, we speculate that patients carrying the *ABCB1* rs2032582 G allele experience lower atorvastatin efflux activity from the hepatocytes into bile and higher hepatocellular concentrations of atorvastatin than carriers of the *ABCB1* rs2032582 T/A alleles. The higher hepatocellular concentration of atorvastatin can increase the risk of hepatotoxicity because atorvastatin induced concentration-dependent cytotoxicity in HepaRG cells (Additional file 1: Figure S1).

The *ABCB1* rs2032582 allele frequencies in our 444 patients (45.2 %, 37.7 % and 17.1 % for G, T and A alleles, respectively) are consistent with the previous report of 154 Japanese subjects (42.9 %, 40.6 % and 16.6 % for G, T and A alleles, respectively) [23]. The above report revealed that the *ABCB1* rs2032582 G and T/A allele frequencies in a Japanese population were comparable with those in a Caucasian population (42.9 % vs. 50.0 % and 57.2 % vs. 50.0 % for G and T/A alleles, respectively) [23]. Taking into account that no differences were reported in the systemic exposure to atorvastatin between Asian and Caucasian subjects [24], the *ABCB1* rs2032582 allele might be also associated with the risk of AILI in the Caucasian population.

Of the atorvastatin-induced adverse reactions, myopathy is one of the most fatal adverse reactions [25, 26]. No statistically significant difference in AUC and the maximum plasma concentrations was observed between 14 patients with atorvastatin-induced myopathy and 15 healthy controls [27]. However, patients with atorvastatin-induced myopathy showed 2.4- and 3.1-fold higher AUC to atorvastatin lactone and *p*-hydroxy atorvastatin, respectively, compared to controls [27]. Atorvastatin is converted to its corresponding lactone form spontaneously or via glucuronidation mediated by UGT1A1, 1A3 and 1A4 and is metabolized to *p*-hydroxy atorvastatin by CYP3A4/5 [11, 28]. The present association studies showed that known functional SNPs of UGT1A1 and CYP3A4/5 were not associated with AILI. The higher accumulation of atorvastatin in the liver of patients carrying the *ABCB1* rs2032582 G allele may cause hepatotoxicity, rather than those of atorvastatin lactone and *p*-hydroxy atorvastatin, the atorvastatin metabolites generated by UGT1A1 and CYP3A4/5. Therefore, the genetic markers might differ between liver injury and myopathy induced by atorvastatin.

In general, DILI can be divided into dose-dependent and idiosyncratic types [29]. The former is related to the pharmacokinetics and/or pharmacological actions of the drug and the latter is related to immune systems, such as human leukocyte antigen (HLA) in a dose-independent manner. In fact, several HLA alleles showed drug-specific associations with DILI, such as HLA-A*33:03 for ticlopidine and HLA-B*57:01 for flucloxacillin [30]. Therefore, we examined association of HLA alleles with AILI. However, no significant association was observed for HLA-A, -B and -C alleles with AILI (Additional file 1: Table S3, Additional file 1: Table S4 and Additional file 1: Table S5).

## Conclusions

Our results showed that *ABCB1* rs2032582 was associated with an increased risk of AILI in the Japanese population. A genetic test of *ABCB1* rs2032582 may provide useful information for predicting individuals at higher risk of AILI. However, additional studies with larger sample size are needed before applying this genetic marker in clinical practice.

## Abbreviations

AILI, Atorvastatin-induced liver injury; ALP, Alkaline phosphatase; ALT, Alanine aminotransferase; AUC, Area under the plasma concentration versus time curve; CI, Confidence interval; DILI, Drug-induced liver injury; FRT, Flp recombination target; HLA, human leukocyte antigen; LDH, Lactate dehydrogenase; OR, Odds ratio; PCR, Polymerase chain reaction; SNPs, Single nucleotide polymorphisms.

## Additional file

Additional file 1: Fig. S1 Atorvastatin concentration-dependent cytotoxicity on HepaRG cells. Fig. S2 Expression levels of ABCB1 protein in Flp-In-293 cells stably expressing ABCB1 proteins encoded by 2677G wild-type, 2677 T and 2677A alleles. Fig. S3 Cell viability curve for IC50 determination in Flp-In-293 cells stably expressing ABCB1 proteins encoded by 2677G wild-type, 2677 T and 2677A alleles. Table S1 Distribution of disease status in 30 AILI and 414 non-AILI patients registered in BioBank Japan. Table S2 Frequency of rs2032582 in 30 AILI and 414 non-AILI patients. Table S3 Association of HLA-A alleles with atorvastatin-induced liver injury. Table S4 Association of HLA-B alleles with atorvastatin-induced liver injury. Table S5 Association of HLA-C alleles with atorvastatin-induced liver injury. (DOCX 147 kb)
