# Supplementary material for: ABCB1 polymorphism is associated with atorvastatin-induced liver injury in Japanese population
Source: BMC Genet. 2016 Jun 13;17:79. doi: 10.1186/s12863-016-0390-5 (PMC4906899; doi:10.1186/s12863-016-0390-5)
Supplement: Additional file 1: — Fig. S1 Atorvastatin concentration-dependent cytotoxicity on HepaRG cells. Fig. S2 Expression levels of ABCB1 protein in Flp-In-293 cells stably expressing ABCB1 proteins encoded by 2677G wild-type, 2677 T and 2677A alleles. Fig. S3 Cell viability curve for IC50 determination in Flp-In-293 cells stably expressing ABCB1 proteins encoded by 2677G wild-type, 2677 T and 2677A alleles. Table S1 Distribution of disease status in 30 AILI and 414 non-AILI patients registered in BioBank Japan. Table S2 Frequency of rs2032582 in 30 AILI and 414 non-AILI patients. Table S3 Association of HLA-A alleles with atorvastatin-induced liver injury. Table S4 Association of HLA-B alleles with atorvastatin-induced liver injury. Table S5 Association of HLA-C alleles with atorvastatin-induced liver injury. (DOCX 147 kb) [file 12863_2016_390_MOESM1_ESM.docx]

**
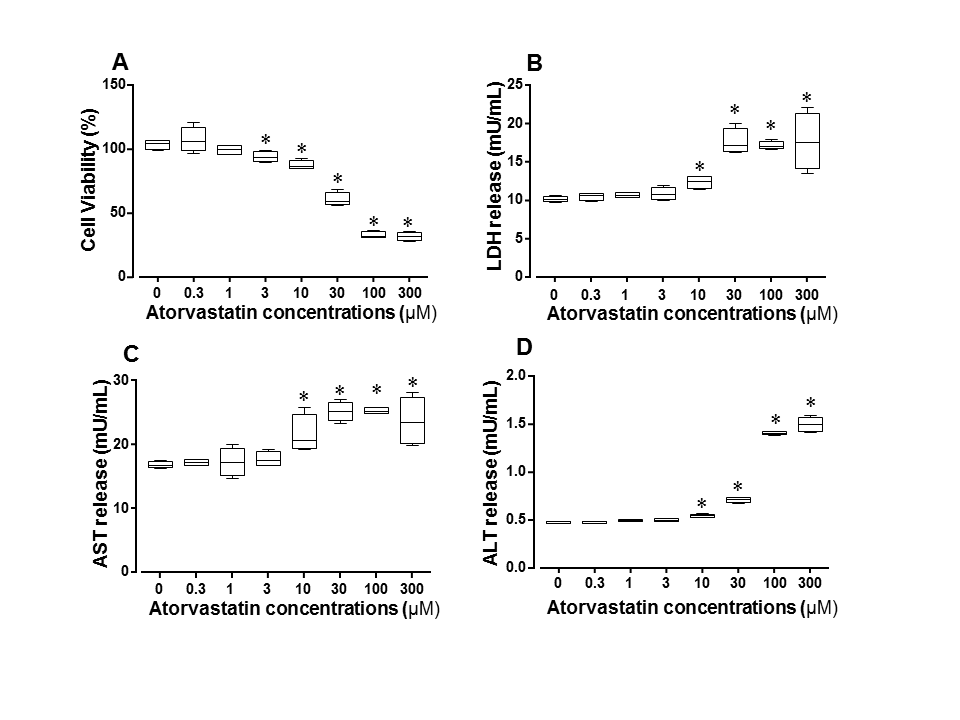
**

**Fig. S1 Atorvastatin concentration-dependent cytotoxicity on HepaRG cells**. Cell viability (A) as well as LDH (B), AST (C) and ALT (D) releases from HepaRG cells into the medium were measured 24 hours after administration of different atorvastatin concentrations. Values are expressed as mean ± standard error of three different experiments performed in duplicates. *P < 0.05, versus 0 µM by a one-way analysis of variance followed with Dunnett's post-hoc test.


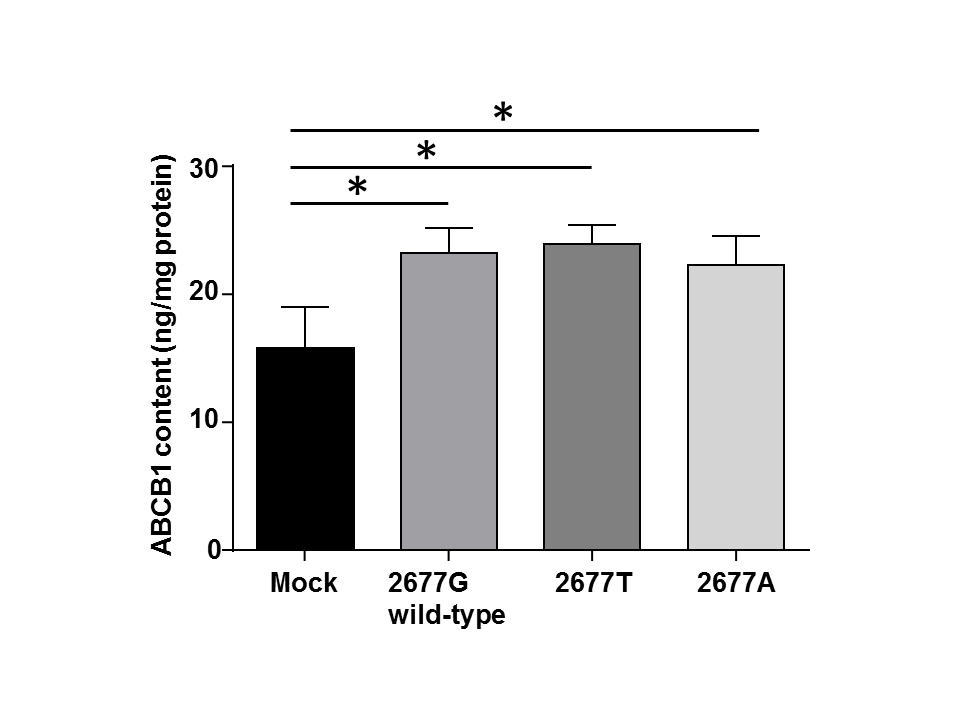


**Fig. S2 Expression levels of ABCB1 protein in Flp-In-293 cells stably expressing ABCB1 proteins encoded by 2677G wild-type, 2677T and 2677A alleles**. Values are expressed as mean ± standard error of three different experiments performed in duplicates. *P < 0.05, versus mock by a one-way analysis of variance followed with Tukey’s post hoc test.

**
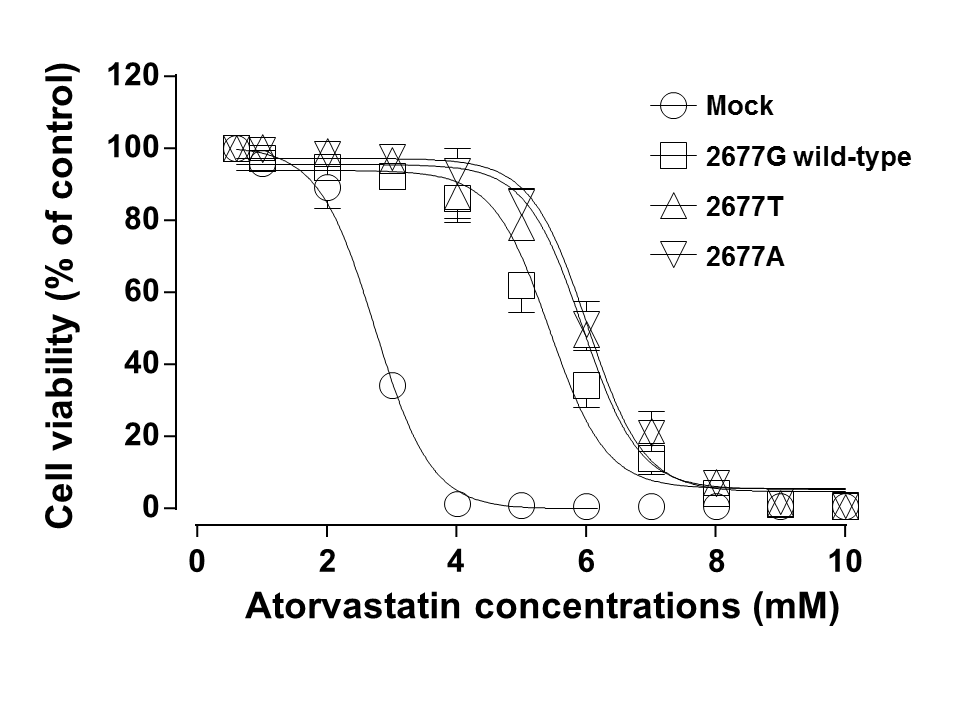
**

**Fig. S3 Cell viability curve for IC50 determination in Flp-In-293 cells stably expressing ABCB1 proteins encoded by 2677G wild-type, 2677T and 2677A alleles**. Values are expressed as mean ± standard error of four different experiments performed in duplicates.

**Table S1 Distribution of disease status in 30 AILI and 414 non-AILI patients registered in BioBank Japan**

| Disease | Number of patients | | P value^b^ |
| --- | --- | --- | --- |
|  | AILI^a^ (%) | Non-AILI^a^ (%) |  |
| Arrhythmia | 1 ( 2.6) | 34 ( 8.2) | 0.50 |
| Arteriosclerosis obliterans (ASO) | 0 ( 0.0) | 10 ( 2.4) | 1.00 |
| Basedow disease, | 1 ( 2.6) | 2 ( 0.5) | 0.19 |
| Breast cancer | 0 ( 0.0) | 4 ( 1.0) | 1.00 |
| Bronchial asthma | 0 ( 0.0) | 5 ( 1.2) | 1.00 |
| Cataract | 2 ( 5.3) | 39 ( 9.4) | 1.00 |
| Cerebral aneurysm | 0 ( 0.0) | 1 ( 0.2) | 1.00 |
| Cervical cancer | 1 ( 2.6) | 32 ( 7.7) | 1.00 |
| Cerebral infarction | 0 ( 0.0) | 1 ( 0.2) | 0.72 |
| Chronic obstructive pulmonary disease (COPD) | 0 ( 0.0) | 1 ( 0.2) | 1.00 |
| Colorectal cancer | 0 ( 0.0) | 4 ( 1.0) | 0.30 |
| Chronic hepatitis B | 0 ( 0.0) | 3 ( 0.7) | 1.00 |
| Chronic hepatitis C | 1 ( 2.6) | 4 ( 1.0) | 1.00 |
| Diabetes mellitus | 12 (31.6) | 102 (24.6) | 0.082 |
| Dyslipidemia | 23 (60.5) | 275 (66.4) | 0.32 |
| Endometrial cancer | 0 ( 0.0) | 1 ( 0.2) | 1.00 |
| Epilepsy | 0 ( 0.0) | 1 ( 0.2) | 1.00 |
| Gastric cancer | 0 ( 0.0) | 3 ( 0.7) | 1.00 |
| Glaucoma | 0 ( 0.0) | 4 ( 1.0) | 1.00 |
| Heart failure | 2 ( 5.3) | 18 ( 4.4) | 0.64 |
| Hematopoietic tumor | 0 ( 0.0) | 1 ( 0.2) | 1.00 |
| Hypersensitivity syndrome (Drug eruption) | 1 ( 2.6) | 0 ( 0.0) | 0.068 |
| Interstital lung disease/Pulmonary fibrosis | 0 ( 0.0) | 1 ( 0.2) | 1.00 |
| Liver cancer | 0 ( 0.0) | 1 ( 0.2) | 1.00 |
| Lung cancer | 0 ( 0.0) | 3 ( 0.7) | 1.00 |
| Myocardial infarction | 5 (13.2) | 73 (17.6) | 1.00 |
| Nephrotic syndrome | 1( 2.6) | 4 ( 1.0) | 0.30 |
| Osteoporosis, | 0 ( 0.0) | 5 ( 1.2) | 1.00 |
| Periodontal disease | 0 ( 0.0) | 1 ( 0.2) | 1.00 |
| Pollinosis | 1 ( 2.6) | 5 ( 1.2) | 0.34 |
| Prostate cancer | 0 ( 0.0) | 2 ( 0.5) | 1.00 |
| Rheumatoid arthritis | 0 ( 0.0) | 5 ( 1.2) | 1.00 |
| Stable angina | 4 (10.5) | 50 (12.1) | 0.77 |
| Tuberculosis | 0 ( 0.0) | 1 ( 0.2) | 1.00 |
| Unstable angina | 5 (13.2) | 21 ( 5.1) | 0.024 |
| Urolithiasis | 0 ( 0.0) | 4 ( 1.0) | 1.00 |
| Uterine fibroid | 0 ( 0.0) | 4 ( 1.0) | 1.00 |

Abbreviation: AILI, atorvastatin-induced liver injury

^a^AILI, *N* = 30; Non-AILI, *N* = 414

^b^The significant P value after Bonferroni correction is less than 0.0014.

**Table S2** Frequency of rs2032582 in 30 AILI and 414 non-AILI patients

|  |  | Number of subjects | | | | | |  | Frequency | | |
| --- | --- | --- | --- | --- | --- | --- | --- | --- | --- | --- | --- |
|  |  | GG | GT | GA | TT | AA | TA |  | G | T | A |
| AILI |  | 12 | 13 | 3 | 1 | 0 | 1 |  | 0.67 | 0.27 | 0.07 |
| Non-AILI |  | 85 | 135 | 56 | 63 | 17 | 58 |  | 0.44 | 0.38 | 0.18 |

Abbreviation: AILI, atorvastatin-induced liver injury

**Table S3 Association of HLA-A alleles with atorvastatin-induced liver injury**

| Allele | Number of carriers | | P value^b^ |
| --- | --- | --- | --- |
|  | AILI^a^ (%) | Non-AILI^a^ (%) |  |
| A*01:01 | 0 ( 0.0) | 5 ( 0.6) | 1.00 |
| A*02:01 | 9 (30.0) | 82 ( 9.9) | 0.24 |
| A*02:06 | 1 ( 3.3) | 87 (10.5) | 0.016 |
| A*02:07 | 4 (13.3) | 24 ( 2.9) | 0.11 |
| A*02:10 | 0 ( 0.0) | 3 ( 0.4) | 1.00 |
| A*02:18 | 0 ( 0.0) | 1 ( 0.1) | 1.00 |
| A*03:01 | 0 ( 0.0) | 4 ( 0.5) | 1.00 |
| A*03:02 | 0 ( 0.0) | 1 ( 0.1) | 1.00 |
| A*11:01 | 7 (23.3) | 70 ( 8.5) | 0.45 |
| A*11:02 | 0 ( 0.0) | 2 ( 0.2) | 1.00 |
| A*24:02 | 20 (66.7) | 218 (26.3) | 0.19 |
| A*24:04 | 0 ( 0.0) | 1 ( 0.1) | 1.00 |
| A*24:08 | 0 ( 0.0) | 1 ( 0.1) | 1.00 |
| A*24:20 | 0 ( 0.0) | 7 ( 0.8) | 1.00 |
| A*26:01 | 3 (10.0) | 68 ( 8.2) | 0.45 |
| A*26:02 | 1 ( 3.3) | 11 ( 1.3) | 0.58 |
| A*26:03 | 3 (10.0) | 19 ( 2.3) | 0.18 |
| A*26:06 | 1 ( 3.3) | 0 ( 0.0) | 0.068 |
| A*30:01 | 0 ( 0.0) | 2 ( 0.2) | 1.00 |
| A*31:01 | 2 ( 6.7) | 82 ( 9.9) | 0.091 |
| A*33:03 | 4 (13.3) | 71 ( 8.6) | 0.80 |

Abbreviation: AILI, atorvastatin-induced liver injury

^a^AILI, *N* = 30; Non-AILI, *N* = 414

^b^The significant P value after Bonferroni correction is less than 0.0024.

**Table S4 Association of HLA-B alleles with atorvastatin-induced liver injury**

| Allele | Number of carriers | | P value^b^ |
| --- | --- | --- | --- |
|  | AILI^a^ (%) | Non-AILI^a^ (%) |  |
| B*07:02 | 2 ( 6.7) | 31 ( 3.7) | 1.00 |
| B*13:01 | 1 ( 3.3) | 10 ( 1.2) | 0.54 |
| B*13:02 | 0 ( 0.0) | 5 ( 0.6) | 1.00 |
| B*15:01 | 9 (30.0) | 60 ( 7.2) | 0.036 |
| B*15:07 | 1 ( 3.3) | 6 ( 0.7) | 0.39 |
| B*15:11 | 1 ( 3.3) | 5 ( 0.6) | 0.345 |
| B*15:18 | 3 (10.0) | 17 ( 2.1) | 0.15 |
| B*15:27 | 1 ( 3.3) | 0 ( 0.0) | 0.068 |
| B*27:04 | 0 ( 0.0) | 4 ( 0.5) | 1.00 |
| B*35:01 | 5 (16.7) | 87 (10.5) | 0.65 |
| B*37:01 | 0 ( 0.0) | 4 ( 0.5) | 1.00 |
| B*38:02 | 1 ( 3.3) | 1 ( 0.1) | 0.13 |
| B*39:01 | 1 ( 3.3) | 31 ( 3.7) | 0.71 |
| B*39:01:02 | 0 ( 0.0) | 1 ( 0.1) | 1.00 |
| B*40:01 | 1 ( 3.3) | 41 ( 5.0) | 0.34 |
| B*40:02 | 1 ( 3.3) | 60 ( 7.2) | 0.10 |
| B*40:03 | 0 ( 0.0) | 2 ( 0.2) | 1.00 |
| B*40:06 | 2 ( 6.7) | 40 ( 4.8) | 0.76 |
| B*40:07 | 0 ( 0.0) | 1 ( 0.1) | 1.00 |
| B*44:02 | 0 ( 0.0) | 2 ( 0.2) | 1.00 |
| B*44:03:01 | 4 (13.3) | 68 ( 8.2) | 0.80 |
| B*44:03:02 | 0 ( 0.0) | 3 ( 0.4) | 1.00 |
| B*46:01 | 4 (13.3) | 33 ( 4.0) | 0.30 |
| B*48:01 | 2 ( 6.7) | 15 ( 1.8) | 0.33 |
| B*51:01 | 4 (13.3) | 53 ( 6.4) | 1.00 |
| B*51:02 | 0 ( 0.0) | 15 ( 1.8) | 1.00 |
| B*52:01 | 7 (23.3) | 84 (10.1) | 0.65 |
| B*54:01 | 6 (20.0) | 68 ( 8.2) | 0.62 |
| B*55:02 | 2 ( 6.7) | 15 ( 1.8) | 0.33 |
| B*55:04 | 0 ( 0.0) | 1 ( 0.1) | 1.00 |
| B*56:01 | 0 ( 0.0) | 10 ( 1.2) | 1.00 |
| B*58:01 | 0 ( 0.0) | 6 ( 0.7) | 1.00 |
| B*59:01 | 1 ( 3.3) | 16 ( 1.9) | 1.00 |
| B*67:01:01 | 0 ( 0.0) | 6 ( 0.7) | 1.00 |
| B*67:01:02 | 0 ( 0.0) | 3 ( 0.4) | 1.00 |

Abbreviation: AILI, atorvastatin-induced liver injury

^a^AILI, *N* = 30; Non-AILI, *N* = 414

^b^The significant P value after Bonferroni correction is less than 0.0014.

**Table S5 Association of HLA-C alleles with atorvastatin-induced liver injury**

| Allele | Number of carriers | | P value^b^ |
| --- | --- | --- | --- |
|  | AILI^a^ (%) | Non-AILI^a^ (%) |  |
| C*01:02 | 13 (43.3) | 121 (14.6) | 0.15 |
| C*10:03 | 0 ( 0.0) | 2 ( 0.2) | 1.00 |
| C*03:02 | 0 ( 0.0) | 7 ( 1.8) | 1.00 |
| C*03:03 | 11 (36.7) | 102 (12.3) | 0.19 |
| C*03:04 | 6 (20.0) | 100 (12.1) | 0.82 |
| C*04:01 | 4 (13.3) | 34 ( 4.1) | 0.31 |
| C*05:01 | 0 ( 0.0) | 3 ( 0.4) | 1.00 |
| C*06:02 | 0 ( 0.0) | 9 ( 1.1) | 1.00 |
| C*07:02 | 4 (13.3) | 84 (10.1) | 0.48 |
| C*07:04 | 2 ( 6.7) | 10 ( 1.2) | 0.19 |
| C*08:01 | 3 (10.0) | 61 ( 7.4) | 0.60 |
| C*08:03 | 0 ( 0.0) | 10 ( 1.2) | 1.00 |
| C*12:02 | 8 (26.7) | 88 (10.6) | 0.49 |
| C*14:02 | 1 ( 3.3) | 54 ( 6.5) | 0.15 |
| C*14:03 | 4 (13.3) | 70 ( 8.5) | 0.80 |
| C*15:02 | 1 ( 3.3) | 28 ( 3.4) | 0.71 |

Abbreviation: AILI, atorvastatin-induced liver injury

^a^AILI, *N* = 30; Non-AILI, *N* = 414

^b^The significant P value after Bonferroni correction is less than 0.0031.
